# Primary laparoscopic RPLND for pure seminona metastasis: feasibility of supine and lateral approaches

**DOI:** 10.1590/S1677-5538.IBJU.2022.0370

**Published:** 2022-08-20

**Authors:** Victor Espinheira Santos, Lucas Fornazieri, Eder Silveira Brazão, Plinio Ramos Pinto, Walter Henriques da Costa, Stênio de Cássio Zequi

**Affiliations:** 1 Departamento de Urologia Hospital A.C Camargo Cancer Center São Paulo SP Brasil Departamento de Urologia, Hospital A.C. Camargo Cancer Center, São Paulo, SP, Brasil

## Abstract

**Introduction:**

Retroperitoneal lymphadenectomy (RPLND) is well established as a primary treatment, especially for high-risk stage I and stage IIA/B nonseminomatous tumors, but its value in seminomatous tumors is underreported (1). Classically, seminomas with isolated retroperitoneal lymphadenopathy are treated with external beam radiation therapy or systemic chemotherapy. Although these modalities are effective, they are associated with significant long-term morbidity (2, 3). Some retrospective studies have demonstrated the potential of RPLND as a first-line treatment for stage IIa seminoma, and two very recent prospective trials, still with interim results: SEMS TRIAL and PRIMETEST(3-7). The RPLND robotic technique has been previously described in the post-chemotherapy scenario, however, surgical videos of primary laparoscopic approach are lacking, especially in seminomatous disease (8).

**Materials and Methods:**

We present two cases of primary videolaparoscopic RPLND, using different approaches.Case 1: Thirty four years-old, with prior right orchiectomy for mixed tumor. After 8 months he presented an two cm enlarged interaortocaval lymph node. Percutaneous biopsy showed pure seminoma metastasis.Case 2: Thirty three years-old, with previous left orchiectomy for stage I pure seminoma, without risk factors. After nine months, the patient had a three cm enlarged para-aortic lymph node.

**Results:**

The surgical time ranged from 150 to 210 minutes, with a maximum bleeding of 300 mL and hospital discharge in 48 hours. In one of the cases, we identified a significant desmoplastic reaction, with firm adhesions to the great vessels, requiring vascular sutures, however, no major complication occurred. Pathological anatomy confirmed pure seminoma lymph node metastases in both cases.

**Conclusion:**

Laparoscopic primary RPLND proved to be technically feasible, with less postoperative pain and early hospital discharge. We understand that more studies should be performed to confirm our oncological results.
